# Impact of gender and mutational differences in hormone receptor expressing non-small cell lung cancer

**DOI:** 10.3389/fonc.2023.1215524

**Published:** 2023-08-28

**Authors:** Robert Hsu, Denaly Chen, Bing Xia, Rebecca Feldman, Wendy Cozen, Luis E. Raez, Hossein Borghaei, Chul Kim, Misako Nagasaka, Hirva Mamdani, Ari M. Vanderwalde, Gilberto Lopes, Mark A. Socinski, Antoinette J. Wozniak, Alexander I. Spira, Stephen V. Liu, Jorge J. Nieva

**Affiliations:** ^1^ Department of Internal Medicine, Division of Medical Oncology, University of Southern California, Los Angeles, CA, United States; ^2^ Norris Comprehensive Cancer Center, University of Southern California, Los Angeles, CA, United States; ^3^ Caris Life Sciences, Phoenix, AZ, United States; ^4^ Division of Hematology-Oncology, Department of Medicine, University of California Irvine School of Medicine, Chao Family Comprehensive Cancer Center, Orange, CA, United States; ^5^ Thoracic Oncology Program, Memorial Cancer Institute/Florida Atlantic University, Pembroke Pines, FL, United States; ^6^ Department of Hematology-Oncology, Fox Chase Cancer Center, Philadelphia, PA, United States; ^7^ Lombardi Comprehensive Cancer Center, MedStar Georgetown University Hospital, Washington, DC, United States; ^8^ Department of Oncology, Barbara Ann Karmanos Cancer Institute, Detroit, MI, United States; ^9^ Department of Medical Oncology, Sylvester Comprehensive Cancer Center at the University of Miami, Miami, FL, United States; ^10^ AdventHealth Cancer Institute, Orlando, FL, United States; ^11^ Hillman Cancer Center, Department of Medicine, Division of Hematology/Oncology, University of Pittsburgh Medical Center, Pittsburgh, PA, United States; ^12^ US Oncology Research, Virginia Cancer Specialists, Fairfax, VA, United States

**Keywords:** gender, hormone receptor, mutational differences, non-small cell lung cancer, disparities

## Abstract

**Background:**

The incidence of lung cancer in the US has been decreasing but a bigger decline has been observed in men despite similar declines in tobacco use between men and women. Multiple theories have been proposed, including exposure to exogenous estrogens. Our study seeks to understand the relationship between hormone receptors (HR), gender, and the genomic landscape of non-small lung cancer (NSCLC).

**Methods:**

3,256 NSCLC tumor samples submitted for molecular profiling between 2013-2018 were retrospectively identified and assessed for HR expression. Hormone receptor (HR+) was defined as ≥ 1% nuclear staining of estrogen receptor-alpha (ER-a) or progesterone receptor (PR) by immunohistochemistry. DNA sequencing by NGS included cases sequenced by the Illumina MiSeq hot spot 47 gene panel (n=2753) and Illumina NextSeq 592 gene panel (n=503). An adjusted p-value (q-value) <0.05 was determined significant.

**Results:**

HR+ was identified in 18.3% of NSCLC. HR+ occurred more commonly in women compared to men (19.6% vs 11.4%, p <0.0001, q <0.0001). *EGFR* mutations occurred more commonly in HR+ NSCLC than HR- NSCLC (20.2% vs. 14.6%, p = 0.002, q=0.007). Overall, men with *EGFR* mutations were affected by HR status with a higher prevalence in HR+ NSCLC while such differences were not seen in women. However, in women ages ≤45, there was a trend towards greater prevalence HR+ NSCLC (25.25% vs. 11.32%, q= 0.0942) and 10/25 (40.0%) of HR+ cases in young women were found to be *EGFR* mutated. *KRAS* mutations and ALK+ IHC expression occurred more in HR+ NSCLC whereas *TP53* mutations occurred more in HR- NSCLC.

**Conclusions:**

Women were more likely to have HR+ NSCLC than men and *EGFR* and *KRAS* mutations occurred more commonly in HR+ NSCLC. Additional studies with more strict inclusion criteria for HR+ are warranted to see if there is benefit to targeting HR in these subgroups.

## Background

Lung cancer is the most common cause of cancer-related deaths in the United States; cigarette smoking is a major risk factor (1). The general incidence of lung cancer has been decreasing in both men and women largely due to decrease in the incidence of smoking, but there has been a minimal decline in the incidence of lung cancer in women (2, 3). Although smoking behaviors are similar between men and women today, historically men had higher prevalence of smoking than women resulting in higher incidence rates of lung cancer. As smoking declined in men, their rates of lung cancer declined precipitously. However, women have not experienced a decline of the same magnitude (4).

Some theories postulate that women have a higher sensitivity to adverse biological effects of smoking including a prevalence of tumor protein *p53* (*TP53*)*/*Kirsten rat sarcoma viral oncogene homolog (*KRAS*) co-mutations, higher levels of polycyclic aromatic hydrocarbons (PAH)-DNA adducts at any given level of smoking, and higher CYP1A1 expression (which encodes an enzyme used in the metabolism of PAHs) (5, 6). Women may have a higher exposure to passive smoking, which is a known risk factor for lung cancer (7). In addition, adenocarcinoma is more common in women especially never-smokers and the risk of adenocarcinoma decreases more slowly than other histologies (8). Consistent with this observation, women have more *EGFR* mutations than men (9). Other non-smoking risk factors for lung cancer include passive smoking (10), viral infections such as human papilloma virus (HPV) (11), low body mass index (BMI) (12), diet (significantly lower grain and carbohydrate consumption in patients with epidermal growth factor receptor (*EGFR*) mutations) (13), socioeconomic status (14), and exposures to arsenic, asbestos and radon (15).

The effect of estrogen on lung cancer pathogenesis is complex and not well understood, and current data linking the effect of estrogen and hormone replacement therapy (HRT) on the incidence of lung cancer is conflicting. Estrogen has two major receptors implicated in carcinogenesis of non-small cell lung cancer (NSCLC): estrogen receptor alpha (ER-a) and estrogen receptor beta (ER-b), both with high affinity for estradiol (16). It has been shown that ER-a, in the presence of estrogen, activates transcription, whereas ER-b inhibits transcription in presence of estrogen (16). Regarding the mechanism of estrogen and the carcinogenesis of lung cancer, some studies show that blocking ER can inhibit proliferation of NSCLC in mice while others have shown estrogen can reduce inflammatory cytokines which reduce the risk of NSCLC. Some have proposed that there may be a protective role with HRT in smokers due to the anti-inflammatory properties of estrogen by neutralizing the extra inflammation induced by smoking (14).

The role of progesterone receptors (PR) in the pathogenesis of NSCLC is unclear and there have been mixed results on the prognostic implications. Ishibashi et al. first looked at PR in NSCLC and showed that PR+ NSCLC was inversely associated with tumor node metastasis (TNM) stage and histology with better clinical outcomes in patients with PR+ status (17). A later study further showed that PR expression in tumor-surrounding stromal cells is associated with improved disease-specific survival and positive PR expression in tumor epithelial cells is associated with poor disease-specific survival in females (18). Yet, Raso et al. did not show any correlation between PR and patient clinicopathologic characteristics, which included histology, gender, tobacco history, and staging (19). We sought in our retrospective study to understand the relationship between HR status, gender, and the genomic landscape in NSCLC.

## Methods

### Tumor samples

The study included NSCLC tumor samples submitted to Caris Life Sciences (Phoenix, AZ) for analysis. This study was conducted in accordance with guidelines of the Declaration of Helsinki, Belmont report, and U.S. Common rule. In keeping with 45 CFR 46.101(b) (4), this study was performed utilizing retrospective, deidentified clinical data. Therefore, this study was considered IRB exempt and patient consent was not required.

### Immunohistochemistry

Immunohistochemistry (IHC) was performed on formalin-fixed paraffin-embedded (FFPE) sections of glass slides. Slides were stained using automated staining techniques, per the manufacturer’s instructions, and were optimized and validated per CLIA/CAO and ISO requirements. HR-positive (HR+) status was defined as ≥ 1+ and ≥ 1% nuclear staining of ER-a (SP1, Ventana) and/or PR (IE2, Ventana) by immunohistochemistry. ALK IHC status was determined using the Ventana ALK CDx Assay (D5F3,Ventana); ALK positivity was defined as 3+ in >1% of cells (20).

### Next-generation sequencing

NGS was performed on genomic DNA isolated from FFPE tumor samples using the NextSeq platform (Illumina, Inc., San Diego, CA). Cases were either sequenced by the Illumina MiSeq hot spot 47 gene panel (n=2753) and Illumina NextSeq 592 gene panel (n=503). For tumors tested with MiSeq, specific regions of the genome were amplified using the Illumina TruSeq Amplicon Cancer Hotspot panel (21, 22). For NextSeq, a custom-designed SureSelect XT assay was used to enrich 592 whole-gene targets (Agilent Technologies, Santa Clara, CA) (23). All variants were detected with > 99% confidence based on allele frequency and amplicon coverage, with an average sequencing depth of coverage of > 500 and an analytic sensitivity of 5%. Prior to molecular testing, tumor enrichment was achieved by harvesting targeted tissue using manual microdissection techniques. Genetic variants were interpreted by molecular geneticists and categorized as “pathogenic,” “presumed pathogenic,” “pathogenic variant”, “variant of unknown significance,” “presumed benign” or “benign” according to the American College of Medical Genetics and Genomics (ACMG) standards. “Pathogenic”, “presumed pathogenic”, and “pathogenic variants” were counted as mutations whereas “benign”, “presumed benign”, and “variants of unknown significance” were excluded. Pan wild type tumors were defined as tumors that did not contain a “pathogenic,” “presumed pathogenic,” or “pathogenic variant” mutation.

### Tumor mutational burden

TMB was measured (592 genes and 1.4 megabases [MB] sequenced per tumor) by counting all non-synonymous missense mutations found per tumor that had not been previously described as germline alterations. TMB analysis was available only for those tumors that were tested with the Illumnia NextSeq 592 gene panel NGS testing.

### Statistical analyses

Standard descriptive statistics were used for this retrospective analysis. For dichotomous outcomes, Fisher’s exact test was performed. For comparison of TMB, student’s t-test was performed. Given the nature of multiple comparisons, p-values with multiple comparisons were further corrected using the Benjamini-Hochberg method and an adjusted p-value (q-value) of <0.05 was considered a significant difference. However, due to the exploratory nature of the investigation, multivariate analysis was not performed, only univariate analysis was performed. Statistical analyses were conducted using R (version 3.5.0) and Prism Graphpad (version 10.0.0).

## Results

### Baseline characteristics

3,256 NSCLC tumor samples submitted for molecular profiling between 2013-2018 were retrospectively identified and assessed for HR expression. There was nearly an even split between males (*n=*1629) and female (*n*=1627) NSCLC tumor samples. The mean age at collection of the sample was 64 years with an average age of 63.75 years in females and 65.08 years in males. By age group, 4.66% (*n*=152) samples were in patients ages ≤ 45, 42.62% (*n*=1388) samples were in patients ages 46-64, and 52.70% (*n*=1716) samples were in patients ages ≥ 65. (**Table 1**).

**Table 1:** Baseline Characteristics of study population.

Total	3256		
Female *n* (%)	1627 (49.96)		
Male *n* (%)	1629 (50.04)		
Age at Collection of Sample	Overall	Female	Male
Mean (SD)	64.41 (10.90)	63.75 (11.37)	65.08 (10.30)
Age ≤45 *n* (%)	152 (4.66)	99 (6.08)	53 (3.25)
Age 46-64 *n* (%)	1388 (42.62)	713 (43.82)	675 (41.44)
Age ≥ 65 *n* (%)	1716 (52.70)	815 (50.09)	901 (55.31)
HR+	*n*	%	
**Total**	504	18.31	
Female	318	19.55	
Male	186	11.42	
Prevalence of Mutations	Overall	Female	Male
*EGFR n* (%)	504 (15.48)	337 (20.71)	167 (10.25)
*KRAS n* (%)	861 (26.44)	503 (30.91)	358 (21.98)
*TP53 n* (%)	1672 (51.35)	779 (47.88)	893 (54.82)
*ERBB2 n* (%)	25 (0.77)	12 (0.74)	13 (0.80)
*BRAF n* (%)	134 (4.12)	84 (5.16)	50 (3.07)
ALK+ IHC *n*/total tested (%)	31/1052 (2.95)	23/502 (4.58)	8/550 (1.45)
TMB	Overall *n=*503	Female *n=*243	Male *n*= 260
	11.01 (8.85)	10.58 (7.44)	11.43 (9.99)

In terms of prevalence of mutations, *TP*53 was most commonly seen in 51.35% of tumor samples followed by *KRAS* (26.44%) and *EGFR* mutations (15.48%). In females, *TP*53 mutations were seen in 47.88% of patients followed by *KRAS* mutations in 30.91% of patients and *EGFR* mutations in 20.71% of patients. In males, *TP*53 mutations were seen in 54.82% of patients followed by *KRAS* mutations in 21.98% of patients, and then *EGFR* mutations in 10.25% of patients. (**Table 1**).

The overall mean TMB was 11.01 mutations/Mb among the 503 patients with TMB tested; the mean TMB was 10.58 mutations/Mb in females (*n*= 243) and 11.43 mutations/Mb in males (*n*=260). (**Table 1**).

### Hormone receptor positivity in NSCLC

Hormone receptor positivity (HR+) was identified in 504/3256 (18.3%) of NSCLC tumors. By gender, HR+ occurred more commonly in women compared to men (19.6% vs 11.4%; p<0.0001, q<0.0001). (**Table 2A**) When stratified by age, women age≥65 were more likely than men age≥65 to have HR+ NSCLC (160/815, 19.6% vs. 95/901, 10.5%; p<0.0001, q<0.0001). In young patients (age ≤ 45), there was a trend towards increased likelihood in women (25/99, 25.3%) compared to men (6/53, 11.3%) (p= 0.0565, q = 0.0942). Among HR+ patients, women had a significantly greater prevalence of ER+ cases (255/318, 80.19% vs.132/186, 70.97%, p = 0.0216, q = 0.0432) while males trended towards having a greater prevalence of PR+ cases (71/172, 38.17% vs. 31.76%, p= 0.1457, q = 0.1457). By estrogen and progesterone receptor positivity, there was a trend towards women having a greater but not statistically significant percentage of ER-a+/PR- prevalence (217/318, 68.24%) compared to men (115/186, 61.83%), (p=0.1457, q= 0.2186) while men trended towards a higher prevalence of ER-a-/PR+ cases (54/186, 29.03% vs. 63/318, 19.81%; p = 0.0216, q = 0.0648) (**Table 2A, B**).

**Table 2:** (A) Hormone receptor status total and percentage in NSCLC by gender and age. (B) Estrogen receptor/progesterone receptor status total and percentage by gender.

A)	HR+	%	HR-	%	Total	p-value (female vs. male)	q-value (female vs. male)		
Total	504	18.31	2752	84.52	3256	<0.0001	<0.0001		
Female	318	19.55	1309	80.45	1627				
Male	186	11.42	1443	88.58	1629				
Age ≤45						p-value (female vs. male)	q-value (female vs. male)		
Female	25	25.25	74	74.75	99	0.0565	0.0942		
Male	6	11.32	47	88.68	53				
Age ≥65						p-value (female vs. male)	q-value (female vs. male)		
Female	160	19.63	655	80.37	815	<0.0001	<0.0001		
Male	95	10.54	806	89.46	901				
**B)**	**ER+/PR+**	**%**	**ER+/PR-**	**%**	**ER-/PR+**	**%**	**Total**	**p-value (**ER+ in female vs. male)	**q-value (**ER+ in female vs. male)
Total									
Female	38	11.95	217	68.24	63	19.81	318	0.0216	0.0432
Male	17	9.14	115	61.83	54	29.03	186		

### EGFR in HR+ NSCLC

**Figure 1:**
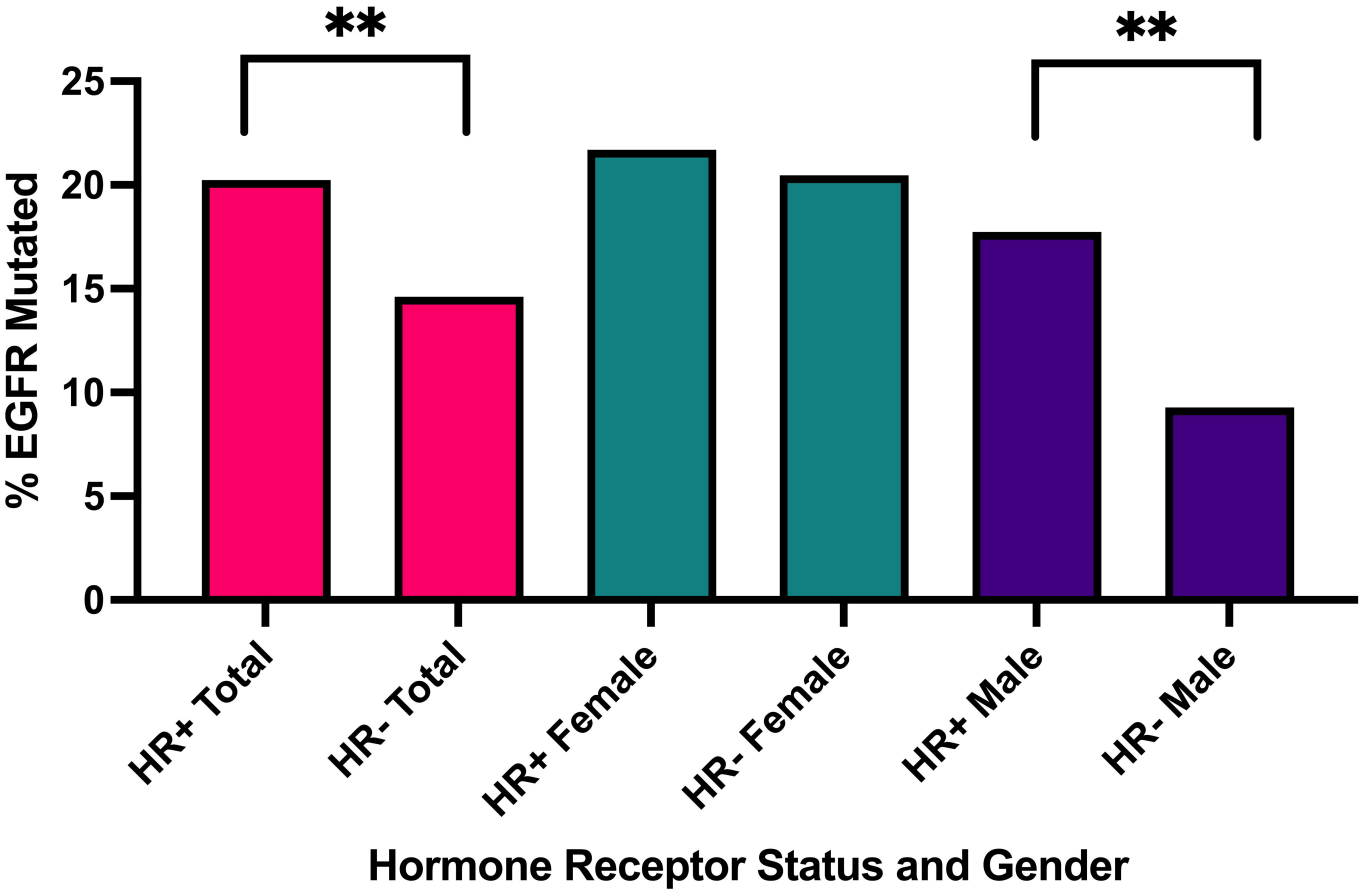
Percentage of *EGFR* mutated cases in all HR+ cases versus HR- cases, in all HR+ female cases versus HR- male cases, in all HR+ male cases versus HR- male cases. **q<0.001.

*EGFR* mutations were observed in 102/504 (20.2%) HR+ NSCLC tumors. *EGFR* mutations occurred more commonly in HR+ NSCLC than HR- NSCLC (102/504, 20.2% vs. 402/2752, 14.6%; p= 0.002, q = 0.007) (**Table 3A**, **Figure 1**). When stratified by gender, men with *EGFR* mutations were affected by HR status with a higher prevalence in HR+ (33/186, 17.7% vs. 134/1443, 9.3%; p = 0.0008, q=0.0056) while there was nearly equal incidence of EGFR mutations in HR+ and HR- females (HR+: 69/318, 21.7% vs. HR-: 268/1309, 20.47%; p =0.6436, q=0.7509).

When further stratified by age, men age ≥ 65 with HR+ NSCLC had significantly greater prevalence of *EGFR* mutations (14/95, 14.74%) compared to HR- NSCLC (p= 0.0124, q=0.0289) while women age ≥ 65 with HR+ NSCLC had similar prevalence of *EGFR* mutations (39/171, 24.38%) compared to HR- NSCLC (121/644, 20.15%) (p=0.2357, q= 0.4125) (**Table 3A**). There was a small sample size of HR+ young patients ≤ 45 years with *EGFR* mutations being tested (*n*= 31), but 10/25 (40.0%) young females had *EGFR* mutations. When we examined estrogen and progesterone receptor positivity in *EGFR* mutants, we observed a similar trend with about 60-65% of both females and males having ER-a+/PR- subtype. However, in *EGFR* wild type patients, females had a greater prevalence of ER-a+ cases (200/249, 80.32% vs. 106/153, 69.28%; p = 0.0157, q= 0.0314). (**Table 3B**).

**Table 3:** (A) Hormone receptor status and *EGFR* mutations in NSCLC total and by gender (B) Estrogen receptor/progesterone receptor status total and percentage by gender.

A)	HR+		HR-		Total	p-value (HR+ vs. HR-)	q-value (HR+ vs. HR-)		
*EGFR*	HR+		HR-		Total				
*EGFR* mutated	102		402		504				
*EGFR* wildtype	402		2350		2752				
%	20.24		14.61		15.48	0.0020	0.0070		
Female	HR+		HR-		Total				
*EGFR* mutated	69		268		337				
*EGFR* wildtype	249		1041		1290				
%	21.70		20.47		20.71	0.6436	0.7509		
Female Age ≤ 45	HR+		HR-		Total				
*EGFR* mutated	10		22		32				
*EGFR* wildtype	15		52		67				
%	40.00		29.72		32.32	0.4584	0.6418		
Female Age ≥ 65	HR+		HR-		Total				
*EGFR* mutated	39		132		171				
*EGFR* wildtype	121		523		644				
	24.38		20.15		20.98	0.2357	0.4125		
Male	HR+		HR-		Total				
*EGFR* mutated	33		134		167				
*EGFR* wildtype	153		1309		1462				
%	17.74		9.29		10.25	0.0008	0.0056		
Male Age ≤ 45	HR+		HR-		Total				
*EGFR* mutated	1		7		8				
*EGFR* wildtype	5		40		45				
%	16.67		14.89		11.86	>0.9999	>0.9999		
Male Age ≥ 65	HR+		HR-		Total				
*EGFR* mutated	14		55		69				
*EGFR* wildtype	81		751		832				
%	14.74		6.82		7.66	0.0124	0.0289		
**B)**	**ER+/PR+**	**%**	**ER+/PR-**	**%**	**ER-/PR+**	**%**	**Total**	**p-value (**ER+ in female vs. male)	**q-value (**ER+ in female vs. male)
*EGFR* Mutated									
Female	10	14.49	45	65.22	14	20.29	69	>0.9999	>0.9999
Male	6	18.18	20	60.61	7	21.21	33		
*EGFR* wildtype									
Female	28	11.24	172	69.08	49	19.68	249	0.0157	0.0314
Male	11	7.19	95	62.09	47	30.72	153		

In HR+ NSCLC, there was no significant difference in the prevalence of *EGFR* mutations (69/318, 21.70% vs. 33/186, 17.74%,; p = 0.3032, q=0.3826) in females. *EGFR* exon 19 deletions were the most common subtype observed at (42/102, 41.18%) followed by *EGFR* L858R mutations (27/102, 26.47%), and exon 20 insertions (9/102, 8.82%). There was also a small percentage of secondary T790M mutations (8/102, 7.84%). Males and females had a similar distribution amongst the *EGFR* mutation subtypes, but it is worth noting that 8 of the 9 *EGFR* Exon 20 insertions in HR+ NSCLC occurred in women. With regards to *EGFR TP53* comutations, there was a suggestive trend towards higher prevalence in women (42/69, 60.87% vs. 15/33, 45.45%, p= 0.2007; q= 0.2007) in HR+ NSCLC. Meanwhile, males had a greater prevalence of *EGFR TP53* comutations in HR- NSCLC compared to HR+ NSCLC (91/134, 67.91% vs. 15/33, 45.45%, p = 0.0254, q= 0.0762). (**Table 4**).

**Table 4:** Hormone receptor status and *EGFR* subtypes by total and by gender.

*EGFR* subtypes	HR+	%	HR-	%	Total	p-value (HR+ vs. HR-)	q-value (HR+ vs. HR-)
Exon 19 del	42	41.18	154	38.31	196	0.6494	>0.9999
L858R	27	26.47	112	27.86	139	0.8056	>0.9999
Exon 20 ins	9	8.82	19	4.73	28	0.1423	>0.9999
G719X|S768I|L861Q	4	3.92	21	5.22	25	0.7991	>0.9999
Uncommon nonclassical mutations	12	11.76	69	17.16	81	0.2273	>0.9999
T790M	8	7.84	27	6.72	35	0.6655	>0.9999
Total	102		402		504		
*EGFR* subtypes Female	HR+	%	HR-	%	Total		
Exon 19 del	27	39.13	99	36.94	126	0.7808	>0.9999
L858R	19	27.54	76	28.36	95	>0.9999	>0.9999
Exon 20 ins	8	11.59	13	4.85	21	0.3599	>0.9999
G719X|S768I|L861Q	3	4.35	15	5.60	18	>0.9999	>0.9999
Uncommon nonclassical mutations	8	11.59	45	16.79	53	0.2273	>0.9999
T790M	4	5.80	20	7.46	24	0.7957	>0.9999
Total	69		268				
*EGFR* subtypes Male	HR+	%	HR-	%	Total		
Exon 19 del	15	45.45	55	41.04	70	0.6959	>0.9999
L858R	8	24.24	36	26.87	44	0.8288	>0.9999
Exon 20 ins	1	3.03	6	4.48	7	>0.9999	>0.9999
G719X|S768I|L861Q	1	3.03	6	4.48	7	>0.9999	>0.9999
Uncommon nonclassical mutations	4	12.12	24	17.91	28	0.6037	>0.9999
T790M	4	12.12	7	5.22	11	0.2304	>0.9999
Total	33		134		167		
*EGFR* TP53 comutation	HR+	%	HR-	%	Total		
Female	42	60.87	149	55.60	191	0.4963	0.5004
Male	15	45.45	91	67.91	106	0.0254	0.0762
Total	57	55.88	240	59.70	297	0.5004	0.5004

### Other mutations in HR+ NSCLC

**Figure 2:**
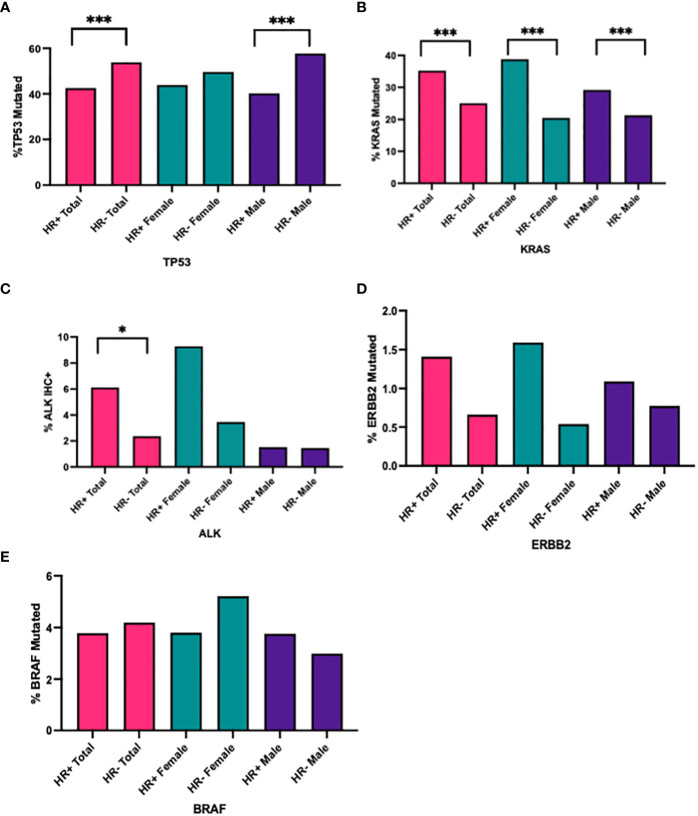
Percentage of **(A)** TP53, **(B)** KRAS, **(C)** ALK+ IHC, **(D)** ERBB2, **(E)** BRAF mutated cases in all HR+ cases versus HR- cases, in all HR+ female cases versus HR- male cases, in all HR+ male cases versus HR- male cases. ***q<0.0001, *q<0.05.

There was a significantly higher prevalence of *TP53* mutations in HR- NSCLC vs. HR+ NSCLC (53.89% vs. 42.54%; p<0.0001, q <0.0001); larger differences were seen in males (HR-: 57.72% vs. HR+ 40.22%; p<0.0001, q<0.0001) compared to females (HR-: 49.69% vs. HR+: 43.91%, p=0.0676, q = 0.1082) (**Table 5**, **Figure 2A**).

**Table 5:** Hormone receptor status and *KRAS, TP53*, ALK IHC+, *BRAF*, and *ERBB2* mutations by total and by gender.

	HR+		HR-		Total	p-value (HR+ vs. HR-)	q-value (HR+ vs. HR-)
TP53	HR+		HR-		Total		
*TP53* mutated	211		1461		1672		
*TP53* wildtype	285		1250		1535		
%	42.54		53.89		52.14	<0.0001	<0.0001
Indeterminate	8		41		49		
Female	HR+		HR-		Total		
*TP53* mutated	137		642		779		
*TP53* wildtype	175		650		825		
%	43.91		49.69		48.57	0.0676	0.1082
Indeterminate	6		17		23		
Male	HR+		HR-		Total		
*TP53* mutated	74		819		893		
*TP53* wildtype	110		600		710		
%	40.22		57.72		55.71	<0.0001	<0.0001
Indeterminate	2		24		26		
KRAS	HR+		HR-		Total		
*KRAS* mutated	177		684		861		
*KRAS* wildtype	325		2049		2374		
%	35.26		25.03		26.62	<0.0001	<0.0001
Indeterminate	2		19		21		
Female	HR+		HR-		Total		
*KRAS* mutated	123		268		391		
*KRAS* wildtype	194		1041		1235		
%	38.80		20.47		24.05	<0.0001	<0.0001
Indeterminate	1				1		
Male	HR+		HR-		Total		
*KRAS* mutated	54		304		358		
*KRAS* wildtype	131		1124		1255		
%	29.19		21.29		22.19	<0.0001	<0.0001
Indeterminate	1		15		16		
*KRAS TP53* comutations		%		%			
Female	50	40.65	145	54.10	195		
Male	19	35.19	122	40.13	141	<0.0001	0.0165
ALK	HR+		HR-		Total		
ALK IHC+	10		21		31		
ALK IHC-	153		868		1021		
**%**	6.13		2.36		2.95	0.0190	0.0434
Unknown	341		1863		2204		
Female	HR+		HR-		Total		
ALK IHC+	9		14		23		
ALK IHC-	88		391		479		
**%**	9.28		3.46		4.58	0.0259	0.0518
Unknown	221		904		1125		
Male	HR+		HR-		Total		
ALK IHC+	1		7		358		
ALK IHC-	65		477		1255		
**%**	1.52		1.45		22.19		
Indeterminate	120		959		1079	>0.9999	>0.9999
	**HR+**		**HR-**		**Total**		
ERBB2	HR+		HR-		Total		
*ERBB2* mutated	7		18		25		
*ERBB2* wildtype	491		2701		3192		
**%**	1.41		0.66		0.78	0.0937	0.1363
Indeterminate	6		33		39		
Female	HR+		HR-		Total		
*ERBB2* mutated	5		7		12		
*ERBB2* wildtype	310		1290		1600		
**%**	1.59		0.54		0.74	0.0659	0.1082
Indeterminate	3		12		15		
Male	HR+		HR-		Total		
*ERBB2* mutated	2		11		13		
*ERBB2* wildtype	181		1411		1592		
**%**	1.09		0.77		0.81	0.6525	0.7457
Indeterminate	3		21		24		
	**HR+**		**HR-**		**Total**		
BRAF	HR+		HR-		Total		
*BRAF* mutated	19		115		134		
*BRAF* wildtype	483		2627		3110		
**%**	3.78		4.19		4.13	0.8070	0.8608
Indeterminate	2		10		12		
Female	HR+		HR-		Total		
*BRAF* mutated	12		72		84		
*BRAF* wildtype	304		1309		1613		
**%**	3.80		5.21		4.95	0.3875	0.5167
Indeterminate	2		6		8		
Male	HR+		HR-		Total		
*BRAF* mutated	7		43		50		
*BRAF* wildtype	179		1396		1575		
**%**	3.76		2.99		3.07	0.5022	0.6181
Indeterminate	0		4		4		

There was a higher prevalence of *KRAS* mutations in HR+ NSCLC (35.26% vs. 25.03%, p<0.0001, q <0.001), seen in both females (HR+: 38.80% vs. HR- 20.47%, p<0.0001, q<0.0001) and males (HR+: 29.19% vs. HR-: 21.29%, p<0.0001, q <0.0001) (**Table 5**, **Figure 2B**). Interestingly, when looking at *KRAS* and *TP53* comutations, there were higher percentage of *KRAS* and *TP53* comutations in HR- NSCLC, particularly in females (HR+: 40.65% vs. HR-: 54.10%, p<0.0001, q =<0.0001) (**Table 5**).

In samples tested for ALK IHC expression (n=1052), there was an increased proportion of ALK IHC expression in HR+ NSCLC compared with HR- (10/163, 6.13% vs. 21/889, 2.36%, p = 0.0190, q =0.0434). When stratified by gender, we saw a trend towards increased prevalence of ALK+ IHC in HR+ NSCLC versus HR- NSCLC in females (9/97, 9.28% vs. 14/405, 3.46%, p = 0.0259, q=0.0518) but not in males (1/66, 1.52% vs. 7/484, 1.45%, p>0.9999, q >0.9999) (**Table 5**, **Figure 2C**).

We did not see a significant difference in HR+ and HR- NSCLC in receptor tyrosine-protein kinase erbB-2 (*ERBB2*) and v-raf murine sarcoma oncogene homolog B1 (*BRAF*) mutations in our population (**Table 5**, **Figures 2D, E**).

### Prevalence of mutations in NSCLC cases ages ≤ 45

There were 152 NSCLC cases in our study population in which the age of collection was 45 years old or younger. Given the small sample size, we were not able to detect statistically significant differences, but we saw that females had a higher frequency of *EGFR* mutations overall (32/99, 32.32% vs. 8/53, 15.09%) and seen in both HR+ NSCLC (10/25, 40.00% vs. 1/6, 16.67%) and in HR- NSCLC (22/74, 29.73% vs. 7/47, 14.89%). Furthermore, there appeared to be a greater percentage of ALK+ IHC cases in females (4/22, 18.18% vs. 1/19, 5.26%), but very small sample sizes of ALK+ tested in HR+ (*n*= 6). (**Table 6**)

**Table 6:** Prevalence of mutations in patients age ≤45 by gender and by hormone receptor status.

Prevalence of Mutations in age ≤45	Overall, *n*=152	Female, *n*=99	Male, *n*=53	HR+ Female, *n*=25	HR+ Male, *n*=6	HR- Female, *n*= 74	HR- Male, *n =* 47
*EGFR n* (%)	40 (26.32)	32 (32.32)	8 (15.09)	10 (40.00)	1 (16.67)	22 (29.73)	7 (14.89)
*KRAS n* (%)	23 (15.13)	15 (15.15)	8 (15.09)	3 (12.00)	2 (33.33)	12 (16.21)	6 (12.77)
*TP53 n* (%)	80 (52.63)	48 (48.48)	32 (60.38)	8 (32.00)	2 (33.33)	40 (54.05)	30 (63.83)
*ERBB2 n* (%)	4 (2.63)	2 (2.02)	2 (3.77)	0 (0.00)	0 (0.00)	2 (2.7)	2 (4.26)
*BRAF n* (%)	3 (1.97)	1 (1.01)	2 (3.77)	0 (0.00)	0 (0.00)	1 (1.35)	2 (4.26)
ALK+ IHC *n*/total tested (%)	5/41 (12.20)	4/22 (18.18)	1/19 (5.26)	1/6 (16.67)	None tested	3/16 (18.75)	1/19 (5.26)

### TMB analysis

The overall mean TMB was 11.01 mutations/Mb among the 503 patients with TMB tested; the mean TMB was 10.58 mutations/Mb in females (*n*= 243) and 11.43 mutations/Mb in males (*n*=260). In HR+ NSCLC, the mean TMB was 9.88 mutations/Mb while in HR- NSCLC the mean TMB was 11.20 mutations/Mb (p=0.2550, q=0.3385). By gender, females trended towards a higher TMB in HR+ NSCLC (10.88 mutations/Mb vs. 8.27 mutations/Mb, p = 0.0891, q= 0.2163). (**Table 7**)

**Table 7:** Tumor Mutational Burden by gender and in *KRAS* mutant cases.

TMB	HR+		p-value/q-value (female vs. male)	HR-		p-value/q-value (female vs. male)	Overall		p-value/q-value (female vs. male)	p-value/q-value (HR+ vs. HR-)	
Overall Mean (SD)	9.88 (5.82)	*n*=68		11.20 (9.23)	*n*=435		11.01(8.85)	*n=* 503	0.2821/ 0.3385	0.2550/ 0.3385	
Male	8.27 (4.65)	*n*=26	0.0721/ 0.2163	11.78 (10.36)	*n*=234	0.1545/ 0.3090	11.43 (9.99)	*n=*260		0.0891/ 0.2163	
Female	10.88 (6.29)	*n*=42		10.52 (7.67)	*n*=201		10.58 (7.44)	*n*=243		0.7740/ 0.7740	
TMB in KRAS mutants										
*KRAS* mutants overall	11.17 (4.70)	*n=*23		10.21 (5.66)	*n*=109		10.37 (5.50)	*n*=132	0.1971/ 0.3942	0.4478/ 0.7165
Female	12.88 (4.5)	*n=*16	0.0056/ 0.0448	10.38 (6.02)	*n*=63	0.7156/ 0.7964	10.88 (5.81)	*n*=79		0.1259/ 0.2957
Male	7.28 (2.29)	*n*=7		9.98 (5.16)	*n*=46		9.62 (4.97)	*n*=53		0.1843/ 0.3942
*KRAS TP*53 comutated	12.75 (5.42)	*n*=12		12.20 (6.70)	*n*=44		12.32 (6.40)	*n*=56		0.7964/ 0.7964
*KRAS* mutant with no *TP*53 mutation	9.45 (3.17)	*n=*11		8.86 (4.40)	*n=*65		8.94 (4.26)	*n*=76		0.6704/ 0.7964

In our *KRAS* mutant NSCLC cases, the mean TMB was 11.17 mutations/Mb in HR+ cases versus 10.21 mutations/Mb in HR- cases (p=0.4478, q = 0.7165). Specifically by gender, females had a statistically significantly higher TMB (12.88 mutations/Mb vs. 7.28 mutations/Mb, p = 0.0056, q = 0.0448). There were no significant differences in TMB in *KRAS TP*53 co-mutated cases in HR+ and HR- nor in *KRAS* mutant cases with no *TP*53 co-mutation. (**Table 7**)

## Discussion

In our study, we found that a higher percentage of women have lung cancers that are hormone receptor positive and that among hormone receptor positive NSCLC patients, women had a significantly greater prevalence of ER-a positivity. Preclinical studies have examined ER and *EGFR* simultaneously and have found that estrogen through its receptor can stimulate lung cancer cell proliferation, resistance to cell death, angiogenesis, and metastasis (24). Epidemiological evidence is lacking. In a study in the Women’s Health Initiative (WHI) there was no statistically significant association between HRT and the incidence of NSCLC (25). However, no similar investigation has been conducted with hormone based oral contraceptives. Clearly more observational research is needed to conclusively address this question.

We found that HR+ was associated with increased prevalence of *EGFR* mutations in NSCLC patients age≥65 and in males overall, which is interesting as patients with *EGFR* mutant lung cancer typically have a lower median age than the average age of U.S. lung cancer patients and seen more in females (26). Both estrogen signaling and *EGFR* signaling can promote proliferation by inducing tumor angiogenesis through vascular endothelial growth factor (*VEGF*) secretion and other growth factors (27). *EGFR* signaling activation increases the expression and activity of aromatase in NSCLC cells and estrogen can induce epidermal growth factor (*EGF*) production and activate *EGFR* signaling (24). Studies have shown a correlation between both ER-a and ER-b expression and the presence of *EGFR* mutations (19, 28). Further studies are needed to better understand the role of estrogen in older men, but it has been shown that among male patients with advanced NSCLC, those with high serum levels of free β-estradiol had significantly worse survival than those with lower β-estradiol so hormone therapy targeting β-estradiol may have benefit in older men (29).

Meanwhile in women, we did not see significance difference in prevalence of *EGFR* mutations in HR+ versus HR- cases and in women age ≥ 65 years. However, we saw a noticeably higher percentage of females ≤ 45 years with *EGFR* mutations with an even higher percentage (10/25, 40.0%) seen in HR+ NSCLC. In addition, females ≤ 45 years trended towards having higher prevalence of HR+ NSCLC compared to males. Young lung cancer patients have a different profile, as many young lung cancer patients are never smokers, have actionable mutations (most common being *ALK* and *EGFR*), and have predominantly adenocarcinoma histology (30, 31). Comparing between young women and men, the lung cancer incidence in young women has been more rapid than the incidence in young men with much of this driven by increases in adenocarcinoma incidence rates in women (32). Much of the reasoning for this remains unclear but strong family genetics may play a role in lifetime nonsmoking women being more suspectable to lung cancer (33). Plus, research has shown that female sex, age of diagnosis ≤ 60, and those with a family history of cancer had lower DNA repair capacity so further understanding of DNA repair genes beyond *BRCA* may identify targets driving these increases (34).

Our study reflects these patterns, but also shows that HR+ NSCLC and HR+/*EGFR* mutated NSCLC are more common in young women. Comparisons between premenopausal and postmenopausal NSCLC women have shown that that adenocarcinoma is more prevalent in premenopausal women (35). In premenopausal women, estrogens are produced by their ovaries through ER-a and thus targeting ER-a may help aid in the treatment of lung cancer in young women (28).

Various hormonal markers and their association with NSCLC clinical outcomes have been previously investigated. High levels of circulating estrogen have been associated with worse survival both in women and men (36). Overexpression of aromatase leads to poor survival in postmenopausal women with NSCLC (37). ER-b overexpression has been shown to be a predictive factor of poor survival in women particularly when co-expressed with aromatase (38, 39). As our data shows higher prevalence of HR+ with *EGFR* mutations in older age NSCLC patients, future studies directed towards response to TKIs based on aromatase levels and specific ER receptor expression is warranted. Also, since the time period of our study, there have been new novel treatments in patients with *EGFR* Exon 20 insertions and given that 8 of our 9 HR+ *EGFR* Exon 20 insertion cases were female, it may be worth investigating the role of ER+ specifically with *EGFR* Exon 20 insertions (40, 41).

Our study also showed that *TP53* mutations were negatively associated with the presence of hormone receptors. This could be in part because the estrogen receptor positive tumors were more likely to be *EGFR* mutated and this subtype is less commonly associated with *TP53* mutations. Smoking has been associated with *TP53* mutations and not with *EGFR* associated cancers (42). However, *EGFR* and *TP53* co-mutations were seen at a similar prevalence in HR+ and HR- NSCLC, but they were more prevalent in females in our HR+ NSCLC population. *TP53* co-mutation with *EGFR* has conferred worse overall survival to first line *EGFR* TKI use in real world settings and our gender disparity findings in HR+ NSCLC suggest that further investigation is needed (43).

Multiple driver mechanisms and the impact of co-mutations has become increasingly recognized in NSCLC. We showed a significant prevalence in ALK IHC positive NSCLC in HR+ NSCLC compared to HR- NSCLC; this combination has not been studied much in lung cancer and may be worth further investigation first by evaluating HR+ in *ALK* fusion NSCLC. Our study also showed a significant increase in *KRAS* mutations in HR+ NSCLC yet a significant decrease in *KRAS TP53* co-mutations in HR+ NSCLC. Almotlak et al. showed in ER-b/*KRAS* mutant mice models that the combination of an ER-b blocker, fulvestrant, with a pan-HER tyrosine kinase inhibitor dacomitinib had a synergistic anti-tumor effect in treating ER-b positive lung cancer. Furthermore, they showed that sequential immunotherapy improved treatment response, suggesting that this combination may provide a novel approach for HR+ *KRAS* mutated NSCLC (44, 45). On further analysis incorporating TMB analysis, we saw a trend towards lower TMB in HR+ NSCLC but we saw a trend towards higher TMB in females in HR+ NSCLC and that HR+ *KRAS* mutant females specifically had a significantly higher TMB in comparison to males. *KRAS* G12C mutations, which have therapeutic implications, are more seen in women with a younger median age and less of a smoking history (5, 46). As we see that *KRAS* mutant women in HR+ NSCLC have significantly higher TMB but not in HR- NSCLC compared to men, there may be additional benefit incorporating hormone therapy in this subset. Future studies also evaluating *KRAS* mutant subtype, as never smokers are more likely to have G>A transition mutations, along with PD-L1 scores and *STK11*/*KEAP1* mutations maybe beneficial in better understanding higher incidence of *KRAS* mutations in HR+ NSCLC.

With regards to the therapeutic implications of our findings, there have been several studies of anti-estrogen therapy in lung cancer particularly looking at *EGFR* mutated NSCLC. Garon et al. conducted a phase II study looking at erlotinib with fulvestrant in advanced stage NSCLC and did not find a significant difference in overall response rate (ORR), progression free survival (PFS), or overall survival (OS) (47). Meanwhile another randomized phase II trial investigating *EGFR*-TKI naïve postmenopausal women with advanced lung cancer combining gefitinib with fulvestrant showed tolerability but did not show PFS benefit (48). However, it should be noted that these two studies did not limit enrollment to patients who were HR+ nor limit enrollment to patients with *EGFR* mutations.

Our study showed that men had a greater prevalence of PR+ overall. Little has been studied regarding anti-progesterone therapy in NSCLC, however, a recent preclinical study showed that PR contains a polyproline domain (PPD) that inhibits NSCLC cell proliferation and has a synergistic effect when given in combination with *EGFR* TKIs while another preclinical study demonstrated that progesterone can inhibit lung adenocarcinoma cell growth via membrane progesterone receptor alpha (49, 50). Further work targeting progesterone receptors should be considered particularly given that 23% of our HR+ NSCLC cases that were ER-a-/PR+.

The strength of our study was the large cohort of 3,256 NSCLC patients available for testing, compared to most other studies with much smaller sample sizes (17, 19, 38, 51). A limitation was that our markers were not directly comparable to other studies. For IHC of ER-a and PR in our study, we used Sp1 transcription factor (SP1) and Calnexin antibody (IE2) respectively and looked at nuclear staining. Other studies have used mouse monoclonal PAI-1 antibody (1D5), anti-estrogen alpha receptor (6F11), or rabbit polyclonal estrogen alpha receptor (HC-20) antibody clone or have not specified when checking for ER-a positivity and mouse anti-progesterone receptor (MAB429) has also been used to evaluate for PR+ (17, 19, 38, 51). Consequently, there have been large variation in detection rates; for example, a review of studies looking at ER-a positivity in NSCLC showed detection rates ranging from 0-97% (51). Another limitation was that our IHC panel only examined ER-a but not ER-b. Like ER-a, studies looking at ER-b have used different antibody clones (Tau antibody (H-150), anti-estrogen receptor beta antibody (14C8), and estrogen receptor beta 1 antibody (PPG5/10)) with varying percentages of detection from 19-98% looking at expression both in the nucleus and cytoplasm (51). Finally, our dataset did not have information on *KRAS* mutation subtypes, PD-L1, or information on *STK11/KEAP1* which may be useful in better understanding the differences in the higher prevalence of *KRAS* mutations in HR+ NSCLC in both genders and HR+ *KRAS* mutant females having a significantly higher TMB than males. Thus, gauging absolute percentages of HR+ between studies should be cautioned and future studies should standardize the IHC being used to evaluate HR+ in NSCLC.

Further clinical trials in the future evaluating HR+ NSCLC with specific mutations should have more specific inclusion criteria regarding hormone positivity. For example, a Phase I trial recently investigating a combination treatment with aromatase inhibitor exemestane and a carboplatin-based therapy for postmenopausal women with advanced NSCLC showed a significant correlation between overall response rate with level of positive aromatase IHC expression (52). Also, in both NSCLC and breast cancer, there have been new novel agents since the previous phase II studies were completed. There are new TKIs not only in *EGFR* but for *ALK* rearrangements and in *KRAS* G12C (46, 53, 54). A new class of selective estrogen receptor degraders has shown promise in ER+/HER-2- breast cancer; a recent phase 3 trial on elacestrant showed significant benefit in patients with *ESR1* mutation versus standard of care and another phase 2 on camizestrant demonstrated superior PFS when compared to fulvestrant (55, 56). As a greater majority of NSCLC has ER-a expression, these new class of endocrine therapies in breast cancer focusing on *ESR1* mutations may hold promise in future studies in HR+ NSCLC (57).Thus, additional clinical trials with more selective inclusion parameters and investigation of new TKIs and estrogen modulator combinations should be investigated in HR+ NSCLC.

## Data availability statement

The aggregate summarized Caris datasets generated during and/or analyzed during the current study can be requested from corresponding author on reasonable request. The deidentified sequencing data are owned by Caris Life Sciences. Qualified researchers can apply for access to these summarized data by contacting Joanne Xiu, PhD (jxiu@carisls.com) and signing a data usage agreement.

## Ethics statement

Ethical approval was not required for the studies involving humans because In keeping with 45 CFR 46.101(b) (4), this study was performed utilizing retrospective, deidentified clinical data. Therefore, this study was considered IRB exempt and patient consent was not required. The studies were conducted in accordance with the local legislation and institutional requirements. The human samples used in this study were acquired from a by- product of routine care or industry. Written informed consent to participate in this study was not required from the participants or the participants’ legal guardians/next of kin in accordance with the national legislation and the institutional requirements.

## Author contributions

RH: Conceptualization, investigation, visualization, supervision, project administration, data curation, methodology, formal analysis, writing–original draft, writing–review and editing. DC: Conceptualization, investigation, data curation, writing–original draft, writing–review and editing. BX: Conceptualization, investigation, data curation, writing–original draft, writing–review and editing. RF: Conceptualization, investigation, data curation, software, formal analysis, writing–original draft,writing–review and editing. WC: Conceptualization, investigation, writing–original draft,writing–review and editing. LR: Investigation, writing—review and editing. HB: Investigation, writing—review and editing. CK: Investigation, writing—review and editing. MN: Investigation, writing—review and editing. HM: Investigation, writing—review and editing. AMV: Investigation, writing—review and editing. GL: Investigation, writing—review and editing. MS: Investigation, writing—review and editing. AJW: Investigation, writing—review and editing. AIS: Investigation, writing—review and editing. SVL: Investigation, writing—review and editing. JJN: Conceptualization, investigation, visualization, supervision, project administration, writing–original draft, writing–review and editing. All authors contributed to the article and approved the submitted version.
